# Skeletal muscle oxygenation during cycling at different power output and cadence

**DOI:** 10.14814/phy2.13963

**Published:** 2019-02-07

**Authors:** Lisha Shastri, Mariana Alkhalil, Claire Forbes, Tina El‐Wadi, Gerrard Rafferty, Koji Ishida, Federico Formenti

**Affiliations:** ^1^ Centre for Human and Applied Physiological Sciences Faculty of Life Sciences and Medicine King's College London London United Kingdom; ^2^ Research Centre of Health Physical Fitness and Sport Nagoya University Nagoya Japan; ^3^ Nuffield Division of Anaesthetics University of Oxford Oxford United Kingdom; ^4^ Department of Biomechanics University of Nebraska at Omaha Nebraska

**Keywords:** Exercise, muscle, NIRS, oxygen

## Abstract

The selection of cadence during cycling may be determined by a number of factors, including the degree of oxygenation in the exercising skeletal muscle. The purpose of this study was to determine the degree of muscle oxygenation associated with different cycling cadences and exercise intensities, and its putative role in the choice of self‐selected cadence during cycling. We recorded cardiopulmonary and metabolic responses to cycling at exercise intensities of 70% and 90% of the ventilatory threshold (*T*_vent_), and used near‐infrared spectroscopy to determine tissue saturation index as a measure of skeletal muscle (*vastus lateralis*) oxygenation. Twelve participants cycled at cadences of 30, 50, 70, 90, and 110 revolutions per minute (rpm), each for 4 min, in a randomized sequence, interspersed with active recovery periods. Despite cardiopulmonary and metabolic responses being greater at 90% than at 70% *T*_vent_, and at 110 rpm compared with lower cadences, *vastus lateralis* oxygenation was not different between the two exercise intensities and five cadences tested. Our results indicate that skeletal muscle tissue saturation index is not substantially affected during cycling for short periods of time at constant, moderate exercise intensity at cadences between 30 and 110 rpm, suggesting that skeletal muscle oxygenation may not be an important negative feedback signal in the choice of self‐selected cadence during cycling at moderate exercise intensity.

## Introduction

Despite bicycles allowing a wide choice of gears, self‐selected cadences are not necessarily the most energy efficient (Hansen et al. 2002; Whitty et al. 2009) particularly in noncyclists. A number of physiological, biomechanical, and psychological factors determine the selection of cadence, with exercise duration and intensity, energy cost, neuromuscular fatigue, joint moments, and comfort all having an effect (Takaishi et al. 1996; Marsh et al. 2000; Whitty et al. 2009). The factors, or interaction of factors responsible for the selection of cadence have been investigated extensively, but are not yet fully understood (Ansley and Cangley 2009; Vercruyssen and Brisswalter 2010).

Cardiorespiratory responses may be important in the selection of cadence. Heart rate (HR), oxygen consumption (*V̇*O_2_), carbon dioxide production (*V̇*CO_2_), and ventilation (*V̇*_E_) all increase with an increase in cadence (Hagberg et al. 1981; Hirano et al. 2015), either while cycling at moderate power outputs (Zoladz et al. 2000), or against no resistance (Tokui and Hirakoba 2007; Formenti et al. 2015).

Afferent feedback signal from skeletal muscle oxygenation may also contribute to the selection of cadence (Amann et al. 2009; Richards et al. 2011). Near‐infrared spectroscopy (NIRS) allows real time, noninvasive investigation of skeletal muscle oxygenation, as measured by the tissue saturation index (TSI) during cycling. TSI provides an overall index of skeletal muscle oxygenation with oxyhemoglobin (OxyHb) and deoxyhemoglobin (HHb) considered indices of oxygen delivery and extraction, respectively (Grassi and Quaresima 2016). It is generally accepted that HHb increases and TSI decreases while cycling at increasing power output associated with increased metabolic rate (Takaishi et al. 2002b; Ferreira et al. 2006; Boone et al. 2016). In contrast, TSI response to increased cadence at a fixed power output is less clear. For example, increasing cadence from 60 to 110 revolutions per minute (rpm) at a constant power output equivalent to 75% of the lactate threshold was associated with a decrease in mean TSI (Skovereng et al. 2016, 2017), while in other studies increasing cadence from 40 to 80 rpm at a power output equivalent to 60% of the maximal oxygen consumption (*V̇*O_2max_) did not alter TSI (Kounalakis and Geladas 2012). An increase in cadence was associated with greater HHb (Skovereng et al. 2016, 2017), no difference in HHb (Ferreira et al. 2006; Kounalakis and Geladas 2012; Zorgati et al. 2013), or reduced HHb (Zorgati et al. 2015). An increase in cadence was also associated with either greater OxyHb (Ferreira et al. 2006; Hirano et al. 2015), no difference in OxyHb (Zorgati et al. 2015), or reduced OxyHb (Kounalakis and Geladas 2012; Skovereng et al. 2016). These apparently contradictory findings may reflect differences in study design (e.g., exercise duration and effect of fatigue) and changes in blood volume, as indicated by total haemoglobin, which were not always reported in full.

The aim of this study was, therefore, to investigate the effect of different pedaling cadences on *vastus lateralis* oxygenation and blood lactate concentration at exercise intensities below the ventilatory threshold (sub‐*T*_vent_) during cycling. We hypothesized that TSI would decrease both at a very low cadence (30 rpm) due to intermittent blood flow caused by the elevated intramuscular pressures, and at a high cadence (110 rpm) due to the associated increase in oxygen consumption.

## Methods

### Ethical approval

The study was approved by the Research Ethics Committee at King's College London (REC Reference Number: LRS‐16/17‐4097) and conformed to the standards outlined in the Declaration of Helsinki. Each participant provided informed written consent before taking part in the experiments.

### Participants

Twelve healthy participants, including 10 sedentary individuals and two amateur club cyclists volunteered to take part in this study. All participants were nonsmokers, free from metabolic, cardiovascular and respiratory conditions. Participants wore light clothing, refrained from consuming alcohol for 24 h and caffeine for 12 h before testing, and were allowed to consume not more than a light meal up to 2 h before testing.

### Experimental protocol

**Figure 1:**
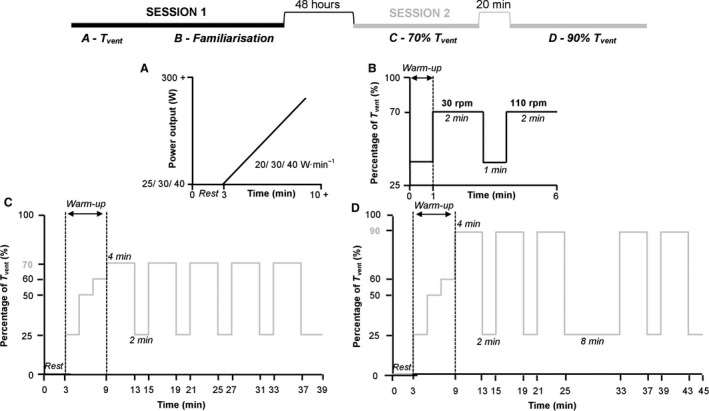
Schematic representation of protocols carried out on sessions one and two of laboratory testing. (A) Identification of *T*_vent_, (B) Familiarization with extreme cadences, (C) 70% *T*_vent_ and D: 90% *T*_vent_ protocols. RPM, revolutions per minute, *T*_vent_, ventilatory threshold.

A schematic diagram illustrating the protocol and its timeline is presented in Figure 1. Participants attended the laboratory on two occasions separated by a minimum of 48 h. Participants’ ventilatory threshold (*T*_vent_) was determined on the first visit, and the responses to cycling at different cadences below *T*_vent_ were tested on the second visit, as described in detail below. The temperature, relative humidity, and atmospheric pressure in the laboratory were 25 ± 2°C, 40 ± 2%, and 758 ± 11 mmHg, respectively (*n* = 30).

#### Ventilatory threshold test and familiarization

The *T*_vent_ was determined using an incremental ramp test on a cycle ergometer. Participants were blind to the power output. The seat height was adjusted appropriately for each participant so that the knee remained slightly flexed at the lowest position of the pedal (Camic et al. 2011; Zuniga et al. 2011); the seat height was recorded and used for each subsequent test. Following a 3‐min rest period, participants started cycling at 70 rpm, a cadence that all participants were expected to be able to sustain and similar to that used in previous investigations (Amann et al. 2004; Zuniga et al. 2011, 2013; Racinais et al. 2014; Formenti et al. 2015). Depending on cycling experience, power output increased from an initial value of 25, 30, or 40 W by 20, 30, or 40 W per minute (W min^−1^) thereby ensuring the total duration of the incremental test was approximately 10 min (Buchfuhrer et al. 1983; Belardinelli et al. 1995). The test was terminated when the respiratory exchange ratio (RER) remained above 1.0 for a period of 90 sec. Muscle oxygenation, HR, and expired gases were recorded continuously. *T*_vent_ was identified as the point at which RER reached 1.0 (Beaver et al. 1986). Further validation of *T*_vent_ was obtained by the *V*‐slope method (Hoogeveen and Hoogsteen 1999), ventilatory equivalent of oxygen method (*V̇*_E_/*V̇*O_2_) (Urhausen et al. 1993), and ventilatory equivalent of carbon dioxide method (*V̇*_E_/*V̇*CO_2_) (Caiozzo et al. 1982). This approach has been previously used (Amann et al. 2004) as it increases the precision of *T*_vent_ estimation compared with a single method (Gaskill et al. 2001).

Following a 10‐min break, a short familiarization test was undertaken, designed to accustom participants with the two extreme cycling cadences (30 and 110 rpm) to be used during the sub‐*T*_vent_ tests. Participants cycled at a power output equivalent to 70% of their *T*_vent_ for 2 min, first at 30 rpm and then at 110 rpm, separated by 1 min of active recovery (70 rpm at 25% *T*_vent_).

#### Physiological responses to different cadences

The main trials consisted of two sub‐*T*_vent_ tests: the first at 70% and the second at 90% *T*_vent_, separated by a break of at least 20 min. These workloads were selected to ensure that participants would be primarily cycling below *T*_vent_, to prevent any drift in V̇O_2_ or other gas exchange parameters also when cadence was elevated to 110 rpm (Skovereng et al. 2016), higher than the 70 rpm cadence used during the incremental test, minimizing recovery time between tests. Both tests commenced with a 3‐min rest period and a 6‐min step‐wise warm up (Fig. 1). The warm up was followed by a series of 4 min cycling bouts at five different cadences (30, 50, 70, 90, and 110 rpm) in a semi‐randomised order: the 110 rpm bout was always performed last (bout five) while the 30 rpm bout was not performed immediately prior to the 110 rpm bout. Due to a limitation of the cycle ergometer in maintaining a constant resistance at cadences lower than 30 rpm, participants were asked to pedal at a cadence just above 30 rpm. Exercise bouts were separated by 2 min of active recovery, during which participants cycled at 25% of *T*_vent_ at 70 rpm, to ensure that TSI and HR returned toward resting values [similar to Takaishi et al. (2002a)]. During the 90% *T*_vent_ test, the active recovery period between the third and fourth exercise bouts was prolonged to 8 min in order to aid recovery of the higher workload.

Blood lactate was sampled in the last 30 sec of rest and of each cycling bout. Expired gases, *vastus lateralis* muscle oxygenation, and HR were recorded continuously. Tests were terminated early if the participant's heart rate exceeded 90% of their theoretical maximum, calculated as 220 ‐ age expressed in years. For the population studied, this equation allowed a marginally greater (up to 2.5%) exercise intensity compared with the equation proposed by Tanaka et al. (2001).

### Measurements and equipment

All cycling tests were performed on an electrically braked cycle ergometer (Corival V3, Lode B.V., Groningen, the Netherlands), with a precision of ±1 W as used in previous investigations [e.g., (Cicchella et al. 2013; Zuniga et al. 2013; Lätt et al. 2016)]. The ergometer was controlled automatically using personalized, pre‐programed protocols within an Oxycon Pro indirect calorimetry system (Jaeger GmnH, Hoechberg, Germany). Cadence and power output were recorded continuously to quantify participants’ adherence to the protocol.

Muscle oxygenation was assessed using a continuous wave near‐infrared spectrometer (PortaMon, Artinis Medical Systems, the Netherlands) similar to the systems used in previous studies (Skovereng et al. 2016, 2017), and described in detail elsewhere (Grassi and Quaresima 2016; Jones et al. 2016). The device contains one receiver and three transmitters, each emitting two wavelengths of 760 and 850 nm. The inter‐optode distance of 35 mm was used for analysis and, using an altered version of the Beer‐Lambert Law and multi‐distance algorithms, TSI and relative concentration changes in OxyHb and HHb were calculated. The amplitude of TSI cyclical oscillations (∆TSI), indicative of dynamic changes in skeletal muscle oxygenation, was also measured and calculated as the difference between the trough and peak of the TSI oscillations within each pedal revolution. The spectrometer was positioned on the muscle belly of the right *vastus lateralis* and secured with an elastic Velcro bandage, minimizing movement of the device and contamination from ambient light during the experiments. NIRS data were sampled at 10 Hz via the online data acquisition system Oxysoft (Artinis Medical Systems, the Netherlands). Double skinfold thickness was determined at the site of application of the NIRS device using the Harpenden Skinfold Caliper (Baty International, UK) and divided by 2 to obtain the actual thickness (Geraskin et al. 2009).

Finger‐prick blood lactate samples were analyzed using a portable lactate meter (Lactate Pro 2, HaB International Ltd., England, UK) (Bonaventura et al. 2015). The system is minimally invasive, reducing impact upon variables being continuously recorded. HR was monitored continuously using a three‐lead electrocardiogram (ECG; HME, Lifepulse, England); ECG data were acquired with analog‐to‐digital sampling (PowerLab 16SP, AD Instruments, Dunedin, New Zealand), recorded and displayed in real time on a computer running LabChart software (version 6, AD Instruments, Dunedin, New Zealand).

Expired gases were analyzed breath‐by‐breath using Oxycon Pro system, as used in previous investigations (Skovereng et al. 2016). This system incorporated a digital triple V‐Volume sensor that allowed the measurements of gas volumes, together with gas analyzers to measure oxygen consumption (*V̇*O_2_) and carbon dioxide production (*V̇*CO_2_). The sensor was connected to a facemask (V Mask, Hans Rudolph, USA), which was checked before testing to ensure a leak‐free fit to the participant's face. The system was calibrated before the beginning of each test for ambient temperature (°C), barometric pressure (mmHg), and relative humidity (%), while respired gas volume and concentration were calibrated using a 3‐L syringe (5530, Hans Rudolph Inc., MO) and standard calibration gases (16% O_2_ and 5% CO_2_), respectively.

### Statistical analysis

Analyses were performed on cadence, power output, HR, blood lactate, *V̇*O_2_, *V̇*CO_2_, TSI, OxyHb, HHb, and ∆TSI. The TSI, OxyHb, and HHb were analyzed as changes from baseline. Mean (±SD) of variables during the *T*_vent_ and sub‐T_vent_ tests were calculated by averaging measurement values from the last 30 sec of each cycling bout. Statistical analyses were performed using Statistical Package for Social Sciences version 20 (SPSS, Inc., Chicago, IL). The normal distribution of variables was checked using the Shapiro‐Wilk test. A paired samples *t*‐test was performed to identify any significant differences in parameters between the 70% and 90% *T*_vent_ conditions for each cadence. Two‐way repeated measures ANOVA corrected for multiple comparisons with Bonferroni's post‐hoc test was performed to identify any significant differences in parameters between cadences within each sub‐*T*_vent_ condition (i. e., within 70% or 90% *T*_vent_). Statistical significance was set at *P *<* *0.05.

## Results

Seven male (58%) and five female (42%) participants completed the study. Participants’ characteristics are summarized in Table 1. The power output at *T*_vent_ was 170 ± 62 W (*T*_vent_ range from 94 to 280 W). Actual rpm during the 5 cycling bouts (30, 50, 70, 90, and 110 rpm) were 34 ± 1, 51 ± 1, 70 ± 1, 89 ± 1, and 108 ± 2 at 70% *T*_vent_ and 35 ± 1, 51 ± 2, 71 ± 2, 89 ± 1, and 109 ± 2 at 90% *T*_vent_.

**Table 1:** Mean and standard deviation values for participants’ characteristics

Parameter	*n* = 12
Age (years)	29 ± 10
Height (m)	1.75 ± 0.09
Weight (kg)	74 ± 11
Skinfold thickness^a^ (mm)	8.0 ± 4.8
Power output at *T*_vent_ (W)	170 ± 62
*V̇*O_2_ at *T*_vent_ (L min^−1^)	2.12 ± 0.74

Participants included 7 male and 5 female individuals. Skinfold thickness was sufficiently small for the NIR light to measure the oxygenation signal in the superficial part of the *vastus lateralis*. The large standard deviation value for the power output at T_vent_ indicates a wide variety of exercise capacity across the participants’ group (*T*_vent_ range from 94 to 280 W).

*T*_vent_, Ventilatory threshold; W, Watt; *V̇*O_2_: oxygen uptake.

a *n* = 10.

### Cardiorespiratory and metabolic function increased with cycling exercise intensity and cadence

**Figure 2:**
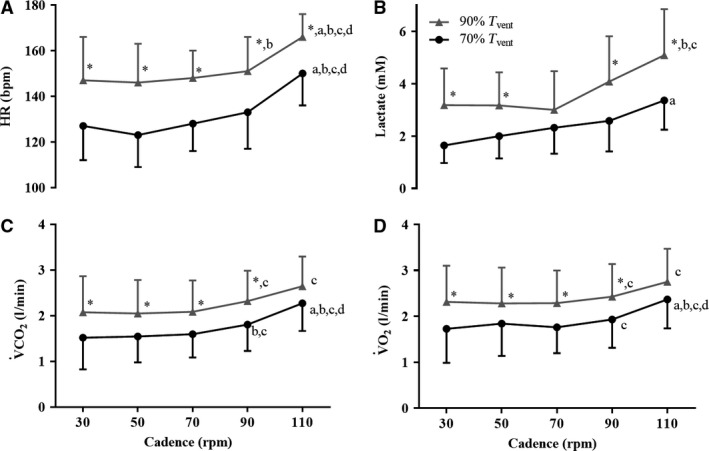
Physiological responses to cycling exercise at different exercise intensities and cycling cadences. Mean and standard deviation values: (A) HR (bpm), (B) Lactate (mM), (C) *V̇*CO_2_ (L·min^−1^) and (D) *V̇*O_2_ (L·min^−1^) for each cadence (30, 50, 70, 90, and 110 rpm) during 70% *T*_vent_ (black symbols) and 90% *T*_vent_ (gray symbols) tests. *N* = 12. *P <* *0.05 when compared to the same cadence at 70% *T*_vent_ using the paired samples *t*‐test. (A) Specific *P*‐values for HR differences between 90% and 70% *T*_vent_ were 0.003, 0.002, 0.002, 0.002 and 0.003 for cadences of 30, 50, 70, 90, and 110 rpm respectively. (B) Specific *P*‐values for blood lactate concentration differences between 90% and 70% *T*_vent_ were 0.006, 0.022, 0.026 and 0.032 for cadences of 30, 50, 90, and 110 rpm. a, b, c, d: *P *<* *0.05 when compared to 30, 50, 70, and 90 rpm respectively, at the same *T*_vent_, using the repeated measures ANOVA with a Bonferroni's post‐hoc correction. HR, heart rate; rpm: revolutions per minute; *T*_vent_, Ventilatory threshold; *V̇* CO_2_, carbon dioxide output; *V̇*O_2_: oxygen uptake.

Figure 2 shows cardiorespiratory and metabolic responses to cycling at different exercise intensities and cadences. HR was significantly higher (~20 beats per minute [bpm]) at 90% *T*_vent_ than at 70% *T*_vent_ at each cadence tested (*P *<* *0.05) (Fig. 2A). Within each exercise intensity, HR at 110 rpm (70% *T*_vent_ = 150 ± 14 bpm and 90% *T*_vent_ = 166 ± 10 bpm) was significantly higher when compared to the lower cadences (*P *<* *0.002).

Blood lactate concentration was significantly higher at 90% than at 70% *T*_vent_ (*P *<* *0.05) (Fig. 2B). Within each exercise intensity, lactate at 110 rpm (70% *T*_vent_ = 3.30 ± 1.16 mmol/L, and 90% *T*_vent_ = 5.30 ± 1.70 mmol/L) was significantly greater when compared to 30 rpm at 70% *T*_vent_ (1.60 ± 0.67 mmol/L, *P *=* *0.003) and when compared to 50 and 70 rpm at 90% *T*_vent_ (3.30 ± 1.34 mmol/L, *P *=* *0.011; 2.90 ± 1.45 mmol/L, *P *<* *0.001).

*V̇*CO_2_ (Fig. 2C) and *V̇*O_2_ (Fig. 2D) were significantly greater at 90% compared to 70% *T*_vent_ at all except the highest cadence tested. *V̇*CO_2_ and *V̇*O_2_ increased significantly at 110 rpm compared with 30, 50, 70, and 90 rpm (*P *<* *0.001) at 70% *T*_vent_, and they were both significantly higher at 90 and 110 rpm compared with 70 rpm at 90% *T*_vent_ (*P *<* *0.05).

### Skeletal muscle oxygenation responses to different cycling exercise intensity and cadence

**Figure 3:**
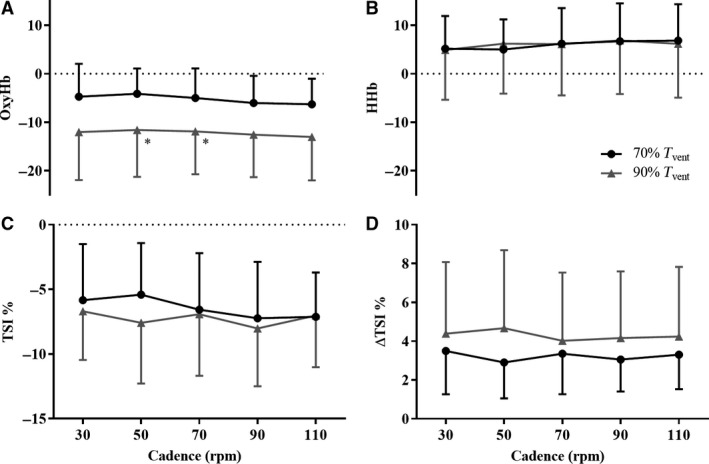
Skeletal muscle oxygenation indices at different exercise intensities and cycling cadences. Mean and standard deviation values: (A) OxyHb, (B) HHb, (C) TSI, (D) ΔTSI for each cadence (30, 50, 70, 90, and 110 rpm) during the 70% *T*_vent_ (black symbols) and 90% *T*_vent_ (gray symbols) tests. *N* = 12. TSI changes from baseline and ΔTSI are presented as percentage values; OxyHb and HHb are presented in arbitrary units and shown as changes from baseline. *P <* *0.05 when compared to the same cadence at 70% *T*_vent_ using the Paired samples *t*‐test. (C) Specific *P*‐values for OxyHb differences between 90% and 70% *T*_vent_ were 0.031 and 0.049 for cadences of 50 and 70 rpm respectively. TSI, Tissue Saturation Index; ΔTSI, amplitude of TSI cyclical oscillation within pedal revolution; Hb, Hemoglobin; OxyHb, Oxygenated Hb; HHb, Deoxygenated Hb; *T*_vent_, Ventilatory threshold; rpm, revolutions per min.

Figure 3 shows *vastus lateralis* oxygenation responses to different exercise intensities and cadences. The OxyHb decrease from baseline was significantly greater at 90% than at 70% *T*_vent_ at cadences of 50 and 70 rpm (*P *<* *0.05), but not significantly different between cadences (Fig. 3A). Changes from baseline in HHb (Fig. 3B) and TSI (Fig. 3C) were not significantly different between 70% and 90% *T*_vent_, and between cadences. An example of the TSI time course during the 90% *T*_vent_ test is presented in the supplementary results for reference. The amplitude of TSI cyclical oscillations (ΔTSI, Fig. 3D) was not significantly different between 70% and 90% *T*_vent_.

## Discussion

This study shows that mean TSI and TSI oscillations within pedal revolution were not different at cycling exercise intensities of 70% and 90% *T*_vent_, and they did not vary in response to changes in cadence.

### Cardiopulmonary and metabolic responses during cycling at different exercise intensities and cadences

We initially hypothesized that the greater cardiopulmonary and metabolic responses at 90% T_vent_ than at 70% T_vent_ could be associated with a smaller TSI, as it is observed during incremental exercise tests (Ferreira et al. 2006; Boone et al. 2015, 2016). Overall, the higher exercise intensity condition (90% *T*_vent_) was associated with a significantly greater cardiorespiratory and metabolic response than the lower exercise intensity condition (70% *T*_vent_). These findings indicate that the 90% *T*_vent_ condition was metabolically more demanding than the 70% *T*_vent_ for most cadences; this difference was limited to a greater heart rate and lactate concentration at 110 rpm, where *V̇*CO_2_ and *V̇*O_2_ responses were not different between the two exercise intensity conditions. These greater cardiorespiratory and metabolic responses associated with an increase in power output from 70 to 90% *T*_vent_ at a constant cadence are in agreement with previous findings (Gaesser and Brooks 1975; Seabury et al. 1977; Hagberg et al. 1981; Grassi et al. 2003), and are particularly evident during incremental exercise (Zoladz et al. 2000; Ferreira et al. 2006; Boone et al. 2015, 2016).

At each exercise intensity, increasing cadence was associated with a greater metabolic response, which reached statistical significance at either 90 or 110 rpm. Increasing cadence while maintaining power output is associated with a greater metabolic cost due to the increased mechanical internal work required to spin the legs, especially at low power output (Formenti et al. 2015), and to the increased work of breathing necessary to sustain the higher minute ventilation. Taken together, these findings indicate that an increase in power output (external mechanical work rate) is a greater determinant of the physiological response to cycling exercise than an increase in cadence (internal mechanical work rate), unless individuals cycle at very low power output levels. From a muscle mechanics perspective, the increase in cadence is associated with a faster shortening velocity, this being three times faster at 90 rpm than at 30 rpm. In the context of our study, these findings suggest that the skeletal muscle operates with a similar efficiency over a range of cadences (i.e., shortening velocities) between 30 and 70 rpm.

Apart from confirming previous findings, these greater cardiopulmonary and metabolic responses observed at 90% compared with 70% *T*_vent_ and at 110 rpm compared with lower cadences were the rationale for exploring skeletal muscle oxygenation levels at different exercise intensities and cadences.

### Skeletal muscle oxygenation responses during cycling at different exercise intensities and cadences

The greater physiological responses at whole body level at 90% than at 70% *T*_vent_ were associated with a significantly greater decrease from baseline in OxyHb (an index of oxygen delivery) in the *vastus lateralis*. OxyHb measurements by NIRS in exercising skeletal muscle can be confounded by changes in skin blood flow, which can increase in order to facilitate heat loss during exercise (Grassi and Quaresima 2016). In contrast with the desaturated exercising skeletal muscle, blood flowing through the skin would be normally saturated during sub‐*T*_vent_ exercise, resulting in an apparently increased recording of skeletal muscle OxyHb. The relatively unchanged laboratory temperature and humidity during each exercise test, together with the limited duration and intensity of the exercise bouts, are unlikely to have determined a large increase in skin blood flow. If an increase in skin blood flow had occurred during our experiments, it was smaller than the decrease in skeletal muscle OxyHb between 70% and 90% *T*_vent_.

There was no significant difference in changes from baseline for HHb and TSI (respectively indices of oxygen extraction and tissue oxygenation) between 70% and 90% *T*_vent_. Similar small changes in HHb were reported in the initial period of constant power output cycling exercise (Hopker et al. 2017), during a ramp test on a cycle ergometer (Boone et al. 2015), and may be in agreement with the relatively limited changes observed in skeletal muscle exercising between lactate threshold and peak oxygen consumption during cycling at similar TSI levels (Ferreira et al. 2006; Formenti et al. 2019).

Cadence did not significantly affect any of the skeletal muscle oxygenation parameters studied, as reported previously (Kounalakis and Geladas 2012; Zorgati et al. 2013) for exercise intensities similar to those considered here. Ferreira et al. (2006) did not observe significant changes in HHb, but did report a marginally greater TSI (~1%) at 100 rpm compared with 60 rpm during 4 min of cycling exercise at 20 W; the physiological importance of this 1% TSI difference appears unclear. An important methodological aspect of these three studies is that cadences were tested in randomised sequence. In contrast, testing cadences in incremental sequence (from 60 to 110 rpm) without recovery or rest periods was associated with a decrease in OxyHb and TSI, and an increase in HHb (Skovereng et al. 2016, 2017) at similar relative power outputs and exercise duration (respectively 75% of the lactate threshold and 4 min) as those considered here (respectively 70% and 90% *T*_vent_ and 4 min). Up to 6 min of exercise at constant power output below lactate threshold is unlikely to result in significant fatigue. It is possible that the changes observed at elevated cadences were the effect of previous exercise at lower cadences, when cadences were tested in incremental sequence and without recovery periods (Skovereng et al. 2016, 2017). We avoided this potentially confounding sequential effect by randomising the cadences tested, and also by adopting periods of active recovery at 25% *T*_vent_. HR and *vastus lateralis*’ TSI before the beginning of each exercise bout returned to values not different from resting levels, suggesting that participants were in similar conditions before the beginning of each exercise bout.

We studied ΔTSI as a dynamic index of skeletal muscle oxygenation within the pedal revolution, expecting larger oscillations in TSI at lower cadences. Here, intramuscular pressure would be greater during contraction, and contraction and relaxation would occur over longer periods of time, possibly resulting in greater dynamic TSI changes. TSI data were sampled (for 30 sec) at 10 Hz, more than twice the minimum required sampling frequency even at the highest cadence of 110 rpm (~1.8 Hz) (Shannon 1948). ΔTSI values averaged ~4% and were not different between 70% and 90% *T*_vent_ and between cadences. This result suggests that the increase in cardiac output and decrease in systemic vascular resistance that occurs during exercise at the intensities studied here (Gotshall et al. 1996) would have contributed to a balanced oxygen delivery‐to‐extraction ratio, the differences in intramuscular pressures, and contraction and relaxation periods not being sufficient to alter TSI within pedal revolution during cycling.

## Conclusion

We conclude that skeletal muscle oxygenation determined by NIRS is not substantially affected during cycling for periods of up to 4 min at sub‐T_vent_ exercise intensity with cadences between 30 and 110 rpm. In this context, our results suggest that skeletal muscle oxygenation may not be an important feedback signal for the choice of self‐selected cadence at moderate exercise intensity.

## Conflict of Interest

The authors report no conflict of interest.
